# Usability of Wearable Multiparameter Technology to Continuously Monitor Free-Living Vital Signs in People Living With Chronic Obstructive Pulmonary Disease: Prospective Observational Study

**DOI:** 10.2196/30091

**Published:** 2022-02-16

**Authors:** Grace Hawthorne, Neil Greening, Dale Esliger, Samuel Briggs-Price, Matthew Richardson, Emma Chaplin, Lisa Clinch, Michael C Steiner, Sally J Singh, Mark W Orme

**Affiliations:** 1 Centre for Exercise and Rehabilitation Science National Institute for Health Research Leicester Biomedical Research Centre – Respiratory University Hospitals of Leicester National Health Service Trust Leicester United Kingdom; 2 Department of Respiratory Sciences University of Leicester Leicester United Kingdom; 3 School of Sport, Exercise and Health Sciences Loughborough University Loughborough United Kingdom

**Keywords:** chronic obstructive pulmonary disease, digital health, physical activity, respiratory rate, wearable technology, wearable device, vital signs monitor

## Abstract

**Background:**

Vital signs monitoring (VSM) is routine for inpatients, but monitoring during free-living conditions is largely untested in chronic obstructive pulmonary disease (COPD).

**Objective:**

This study investigated the usability and acceptability of continuous VSM for people with COPD using wearable multiparameter technology.

**Methods:**

In total, 50 people following hospitalization for an acute exacerbation of COPD (AECOPD) and 50 people with stable COPD symptoms were asked to wear an Equivital LifeMonitor during waking hours for 6 weeks (42 days). The device recorded heart rate (HR), respiratory rate (RR), skin temperature, and physical activity. Adherence was defined by the number of days the vest was worn and daily wear time. Signal quality was examined, with thresholds of ≥85% for HR and ≥80% for RR, based on the device’s proprietary confidence algorithm. Data quality was calculated as the percentage of wear time with acceptable signal quality. Participant feedback was assessed during follow-up phone calls.

**Results:**

In total, 84% of participants provided data, with average daily wear time of 11.8 (SD 2.2) hours for 32 (SD 11) days (average of study duration 76%, SD 26%). There was greater adherence in the stable group than in the post-AECOPD group (≥5 weeks wear: 71.4% vs 45.7%; *P*=.02). For all 84 participants, the median HR signal quality was 90% (IQR 80%-94%) and the median RR signal quality was 93% (IQR 92%-95%). The median HR data quality was 81% (IQR 58%-91%), and the median RR data quality was 85% (IQR 77%-91%). Stable group BMI was associated with HR signal quality (*r*_s_=0.45, *P*=.008) and HR data quality (*r*_s_=0.44, *P*=.008). For the AECOPD group, RR data quality was associated with waist circumference and BMI (*r*_s_=–0.49, *P*=.009; *r*_s_=–0.44, *P*=.02). In total, 36 (74%) participants in the Stable group and 21 (60%) participants in the AECOPD group accepted the technology, but 10 participants (12%) expressed concerns with wearing a device around their chest.

**Conclusions:**

This wearable multiparametric technology showed good user acceptance and was able to measure vital signs in a COPD population. Data quality was generally high but was influenced by body composition. Overall, it was feasible to continuously measure vital signs during free-living conditions in people with COPD symptoms but with additional challenges in the post-AECOPD context.

## Introduction

Chronic obstructive pulmonary disease (COPD) is the third leading cause of death worldwide [1]. People living with COPD may experience an acute exacerbation (ie, acute exacerbation of COPD [AECOPD]), which reduces their quality of life and increases the risk of premature mortality [2].

While often defined by worsening of respiratory symptoms, an AECOPD is associated with changes in heart rate, oxygen saturation [3], and respiratory rate [4], with such vital signs monitored routinely as an inpatient. Transferring this monitoring to the daily lives of outpatients has been challenging but, by doing so, it may be possible to recognize deterioration in health [5,6]. Studies have remotely monitored symptoms through pulse oximetry or spirometry to identify changes in patient health for a while now [7-12], as patients typically find it difficult to identify small day-to-day variations in symptoms [13,14]. The use of remote patient monitoring following hospitalization for an AECOPD is less common [15-18], despite this population being at high risk of readmission to hospital [19,20].

When deploying technological solutions, patient burden is an important barrier to success. To date, studies have relied on patients actively taking daily measurements, such as from pulse oximeters [7,9,10,12,15-17,21,22]. Patient-driven measurements could result in recall bias, errors in data collection [23], and reduced compliance [24-26]. Other studies have required patients to use multiple devices to measure vital signs [12,26,27], which adds to patient burden, with the additional complication of managing multiple devices leading to reduced adherence [26]. It can be even more challenging for individuals following hospitalization for an AECOPD to engage with digital health technologies [17,28,29], perhaps owing to greater disease severity [30]. Providing that it is comfortable and accepted by patients, wearable technology could facilitate free-living health monitoring.

Accordingly, we aimed to determine whether (1) we can measure vital signs using a novel wearable device post hospitalization for AECOPD and during the stable phase of COPD, (2) there are patient characteristics associated with adherence and data quality, and (3) measures of feasibility are different between people post AECOPD and those with stable COPD symptoms.

## Methods

### Recruitment

We performed a prospective, observational cohort study of people living with COPD admitted to hospital for an AECOPD (AECOPD group) and people with stable COPD symptoms (Stable group). This single-center study was undertaken between January 2018 and December 2019 at the University Hospitals of Leicester, the United Kingdom, where individuals were recruited from hospital wards or the pulmonary rehabilitation (PR) service.

People with an AECOPD were screened by COPD specialist nurses and recruited when medically stable and close to being discharged. People with stable disease were screened by the PR team at their initial PR assessment and enrolled prior to starting their PR program. Inclusion criteria were as follows: being ≥18 years old; having a confirmed clinical diagnosis of COPD from spirometry data in medical records; and for the AECOPD group, an admission with a primary diagnosis of exacerbation of COPD was required. Participants were excluded if they had a physical or visual impairment or comorbidities that prevent participation, required palliative care, were participating in another study, or were unable or unwilling to provide written informed consent.

### Vital Signs Measurements Using an Equivital LifeMonitor

All participants were asked to wear an Equivital EQ02+ LifeMonitor device (Equivital) (hereby, “vest”) during waking hours for 6 consecutive weeks. During their baseline visit, participants practiced putting on and removing the vest with a researcher first, and then independently while supervised, and they were also given written and visual instructions to take home. Participants were asked to remove the vest during water-based activities and to charge the sensor electronics module (SEM) overnight, at least every other night.

Our patient and public involvement (PPI) group contributed to the design of the study. Specifically, members selected the Equivital LifeMonitor, from a choice of three wearable devices, and provided feedback on the duration of wear period. They also provided feedback on study documentation, wording, and verbal description of the study for recruitment purposes.

The AECOPD group were asked to start wearing the vest following discharge from hospital, making the day after discharge the first day of wear. The Stable group were asked to wear the vest following their baseline study assessment (after initial PR assessment). The day after the baseline visit was the first day of wear (Multimedia Appendix 1).

Participants were contacted by a researcher via telephone 1-3 days after their baseline visit to evaluate acceptability of the vest. Participants had further follow-up telephone calls on a fortnightly basis for troubleshooting purposes or could contact the study team on an *ad hoc* basis.

The vest measured heart rate (HR), respiratory rate (RR), skin temperature (ST), and physical activity (PA) [31]. PA was classified as stationary or ambulatory using an inbuilt triaxial accelerometer. HR was obtained using built-in ECG electrodes, RR was recorded with a built-in expansion belt, and ST was measured using a thermometer in the SEM (15-second epoch).

### Measures of Feasibility

Definitions and criteria for feasibility indicators are specified in Table 1. Adherence was defined by the number of days the vest was worn during the 6-week study period and the daily wear time. Missing data were examined and classified as either battery depletion (failing to charge the SEM) or nonwear.

The signal quality of HR and RR were based on the proprietary confidence algorithm, accounting for activity and connection artefacts. Based on manufacturer’s recommendations, a signal quality threshold of 85% was used for HR and 80% for RR to indicate whether each 15-second value was deemed acceptable. Data quality for HR and RR was defined as the percentage of daily wear time with acceptable signal quality. Signal quality and data quality were used to identify the confidence of the data generated by the vest.

Field notes from the follow-up phone calls were analyzed to ascertain any common problems that participants had with the technology or other aspects of the study participation. Acceptability was defined as reporting no problems with the technology.

**Table 1:** Measures of feasibility.

Measure (unit)	Definition	Calculation
Duration worn (days)	Number of days that a participant wore the vest across 6 weeks (42 days)	Number of days (out of 42) deemed as worn with minimum wear time thresholds (1-16 hours)
Wear time (hours)	The duration for which the vest was worn in a single day	Sum of the time with a heart rate of >25 beats/min and a skin temperature of >25°C
Heart rate signal quality (%)	The confidence that the heart rate data obtained are accurate	Average heart rate confidence when the vest was worn
Respiratory rate signal quality (%)	The confidence that the respiratory rate data obtained are accurate	Average respiratory rate confidence when the vest was worn
Heart rate data quality (%)	The proportion of daily wear time when the heart rate signal quality was ≥85%	Proportion of time that the heart rate confidence was ≥85% when the vest was worn
Respiratory rate data quality (%)	The proportion of daily wear time when the respiratory rate signal quality was ≥80%	Proportion of time that the respiratory rate confidence was ≥80% when the vest was worn
Skin temperature data quality (%)	The proportion of daily wear time when respiratory rate signal quality or heart rate signal quality was valid	Proportion of time that the respiratory rate confidence was ≥80% or the heart rate confidence was ≥85% when the vest was worn

### Vital Sign Measurements

Vital signs examined were HR, RR, ST, and PA. HR and RR were calculated as the average HR and RR, respectively, during wear time. PA was calculated as the proportion of daily wear time when the patient was ambulatory.

### Participant Characteristics

Demographics, clinical histories, comorbidities, and spirometry data were obtained from medical records or information provided by participants. Height and weight were obtained from medical records or measured. Chest circumference and waist circumference were measured.

The Medical Research Council dyspnea scale [32] was used to measure breathlessness.

### Statistical Analyses

No formal sample size calculation was undertaken for this feasibility study. A sample size of 50 participants per group was decided on the basis of potential suitable participants, logistics, and resources available.

Data were analyzed using R (version 4.0.0). Continuous variables distributions were tested for normality. Data are reported as mean (SD) or median (IQR) and differences between groups were assessed using a 2-sample unpaired *t* test or Mann–Whitney *U* test, respectively. Frequency comparisons between groups were assessed using the Fisher test. The Spearman rank correlation coefficient (*r*_s_) was used to analyze associations between variables (Cronbach α=.05).

### Ethics Approval

All participants provided written informed consent (Research Ethics Committee 15/LO/2055) and the study was prospectively registered (ISRCTN12855961).

## Results

### Recruitment and Participant Characteristics

**Figure 1:**
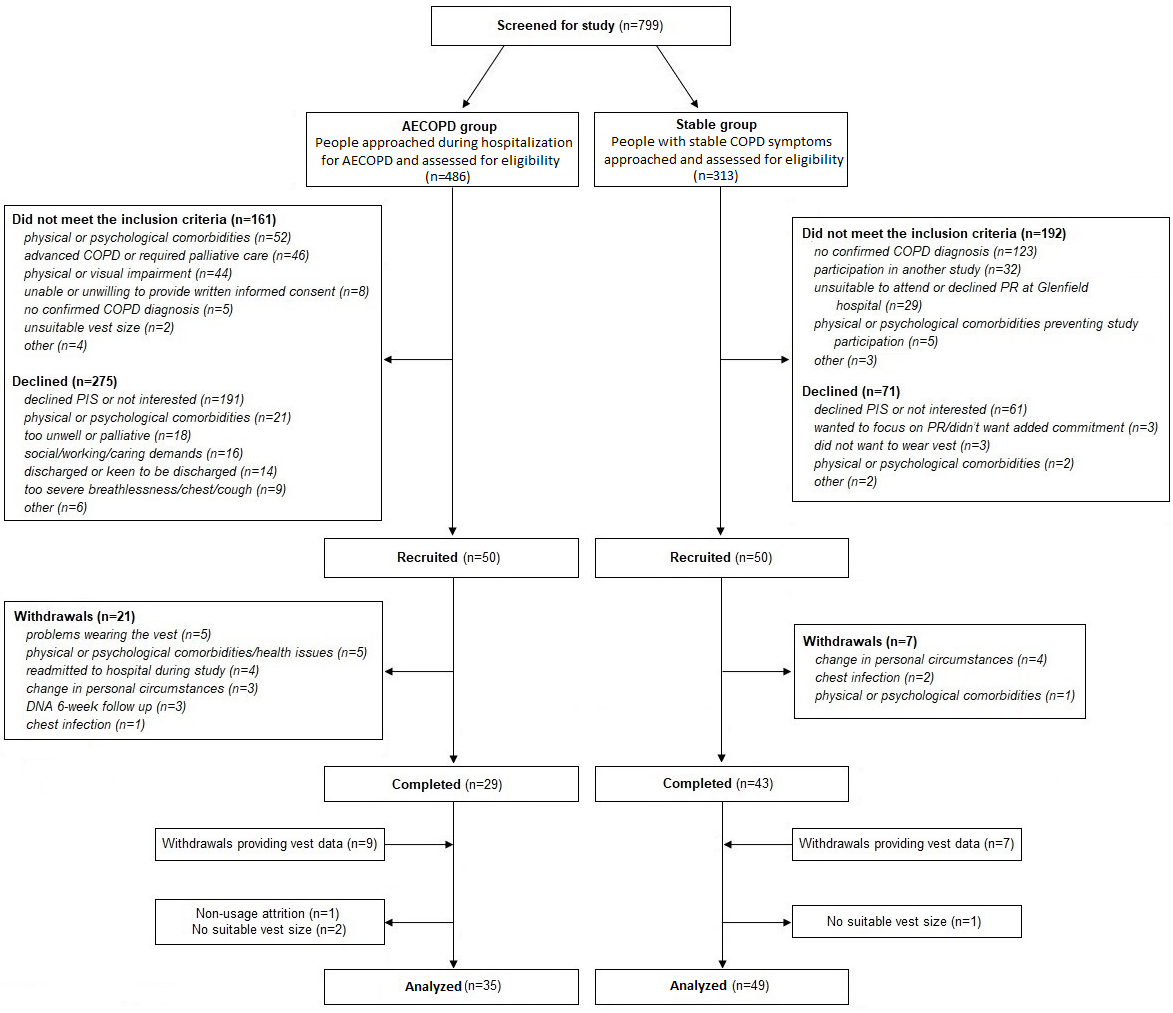
CONSORT (Consolidated Standards of Reporting Trials) flowchart for the AECOPD and Stable groups. AECOPD: acute exacerbation of chronic obstructive pulmonary disease; COPD: chronic obstructive pulmonary disease; DNA: did not attend; PIS: patient information sheet; PR: pulmonary rehabilitation.

Figure 1 outlines recruitment details, reasons for withdrawal, and completion rate for the AECOPD and Stable groups. The AECOPD group had a lower BMI, more severe dyspnea, more hospital admissions, and more frequent exacerbations, but they were otherwise similar to the Stable group (Table 2).

**Table 2:** Baseline characteristics of the AECOPD^a^ and Stable groups.

Characteristics				AECOPD group (n=35)		Stable group (n=49)		*P* value
Male, n (%)				19 (54.3)		27 (55.1)		>.99
Age (years), mean (SD)				67.6 (9.7)		66.7 (9.2)		.77
BMI (kg/m^2^), mean (SD)				25.2 (6.0)		28.0 (6.7)		.04
Chest circumference (inches), mean (SD)				37.9 (4.4)		39.0 (4.0)		.29
Waist circumference (cm), mean (SD)				94.0 (17.1)		97.4 (15.2)		.39
Forced expiratory volume in 1 second (% predicted), mean (SD)				44.8 (22.3)		53.5 (27.4)		.16
Forced expiratory volume in 1 second–forced vital capacity ratio, mean (SD)				0.43 (0.16)		0.50 (0.16)		.14
**Medical Research Council dyspnea grade, n (%)**								
	2		8 (22.9)		15 (30.6)		.47	
	3		5 (14.3)		17 (34.7)		.045	
	4		14 (40.0)		16 (32.7)		.499	
	5		8 (23.8)		1 (2.0)		.003	
**Smoking status, n (%)**								
	Never		0		3 (7.1)^b^		.81	
	Ex-smoker		23 (65.7)		29 (69.1)^b^		.33	
	Current		12 (34.3)		10 (23.8)^b^		.25	
Pack years (years), median (IQR)				48.0 (35.5-63.8)		40.0 (27.0-50.0)^c^		.09
Oxygen use, n (%)				4 (11.4)		3 (7.3)^d^		.70
Hospital admissions in the last 12 months, median (IQR)				1.5 (1.0-2.8)		0 (0-1.0)^e^		<.001
Exacerbations in the last 12 months, median (IQR)				3.0 (2.0-4.0)		0.5 (0-3.0)^e^		.009
Physical activity (hours/day)^f^, median (IQR)				1.3 (0.9-1.7)		1.5 (1.1-2.0)		.03

^a^AECOPD: acute exacerbation of chronic obstructive pulmonary disease.

^b^Missing data (n=7).

^c^Missing data (n=23).

^d^Missing data (n=8).

^e^Missing data (n=25).

^f^Calculated from the Equivital LifeMonitor.

### Feasibility Measures

**Figure 2:**
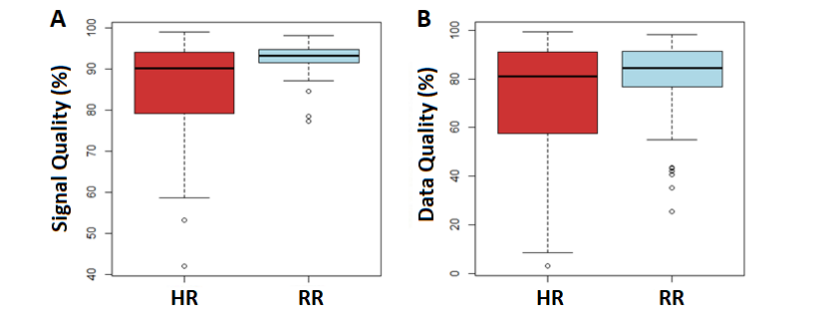
HR and RR (A) signal quality and (B) data quality. Data are shown as box plots composed of the 25th percentile (lower extremity of the box), the median (central line of the box), and the 75th percentile (upper extremity of the box). The lines outside each box correspond to the minimum and maximum values. HR: heart rate; RR: respiratory rate.

For all 84 participants, the vest was worn for a median of 37.0 (IQR 27.8-40.0) days and the median daily wear time was 12.0 (IQR 10.8-13.1) hours. The median HR signal quality was 90% (IQR 80%-94%), and the median RR signal quality was 93% (IQR 92%-95%; Figure 2A). The median HR data quality was 81% (IQR 58%-91%), and the median RR data quality was 85% (IQR 77%-91%; Figure 2B).

There were no significant between-group differences in the number of days the vest was worn, the longest number of consecutive days worn, or the average daily wear time (Table 3). The AECOPD group spent a significantly lesser median time ambulatory (10.1%, IQR 8.6%-15.0% vs 13.4%, IQR 9.5%-19.3%; *P*=.03) and showed a lower median HR signal quality (88.5%, IQR 75.8%-92.6% vs 92.3%, IQR 81.0%-96.4%; *P*=.04) than the Stable group.

**Table 3:** Feasibility and vital sign measures for the AECOPD^a^ and Stable groups.

Measures			AECOPD group (n=35)		Stable group (n=49)		*P* value
Duration worn (days; maximum, 42 days), median (IQR)			33.0 (23.0-39.5)		38.0 (32.0-40.0)		.11
**Duration worn (weeks), n (%)**							
	>5	16 (45.7)		35 (71.4)		.02	
	4-5	8 (22.9)		4 (8.2)		.11	
	3-4	3 (8.6)		5 (10.2)		>.99	
	2-3	2 (5.7)		3 (6.1)		>.99	
	1-2	4 (11.4)		1 (2.1)		.16	
	<1	2 (5.7)		1 (2.1)		.57	
Longest number of consecutive days worn (days), median (IQR)			17.0 (7.0-29.5)		21.0 (10.0-38.0)		.34
Occasions of missing days (days), median (IQR)			2.0 (0.25-5.75)		3.0 (1.0-5.0)		.56
Flat sensor electronics module battery depletion during wear, n (%)			0 (0%)		3 (6%)		.26
Wear time (hours), median (IQR)			12.1 (10.4-12.6)		11.9 (10.9-13.3)		.36
Heart rate signal quality (%), median (IQR)			88.5 (75.8-92.6)		92.3 (81.0-96.4)		.04
Respiratory rate signal quality (%), median (IQR)			93.4 (91.5-94.9)		93.2 (91.8-94.6)		.61
Heart rate data quality (%), median (IQR)			78.1 (55.7-88.0)		86.0 (58.8-95.5)		.10
Respiratory rate data quality (%), median (IQR)			83.7 (75.2-91.5)		85.5 (77.2-89.9)		.97
Skin temperature data quality (%), median (IQR)			95.6 (89.1-97.9)		97.0 (92.1-98.9)		.27
Heart rate (beats/min), mean (SD)			84.4 (10.3)		84.4 (10.2)		.97
Respiratory rate (breaths/min), mean (SD)			20.6 (3.5)		20.3 (3.2)		.71
Skin temperature (°C), mean (SD)			34.2 (0.81)		34.3 (0.98)		.90
Stationary (hours), median (IQR)			10.2 (9.7-11.4)		10.2 (9.5-11.5)		.88
Physical activity (% of wear time), median (IQR)			10.1 (8.6-15.0)		13.4 (9.5-19.3)		.03

^a^AECOPD: acute exacerbation of chronic obstructive pulmonary disease.

### Participant Acceptability

From follow-up phone calls, 21 participants (60%) in the AECOPD group and 36 participants (74%) in the Stable group found the vest acceptable. Five (14%) participants in the AECOPD group reported that they did not wear the vest while unsettled or feeling unwell after returning home. Five (10%) participants in the Stable group and 5 (14%) participants in the AECOPD group experienced some discomfort wearing the vest. Three (9%) participants and 5 (10%) participants in the AECOPD and Stable groups, respectively, reported that they did not wear the vest on days on which they felt unwell. Four (11%) participants in the AECOPD group had problems removing the SEM from the cradle of the vest, compared to one participant (2%) in the Stable group.

### Vest Fitting

For the whole sample, 33 (40%) participants were allocated a larger vest size, which was comparable between groups. Compared to those who completed the study, a larger proportion of participants who withdrew required a larger vest size than the manufacturer’s guidance (71% vs 34%, *P*=.005). There were no associations between participants vest fitting and other feasibility measures (Multimedia Appendix 1).

### Relationships Between Feasibility Measures and Anthropometric Characteristics

**Figure 3:**
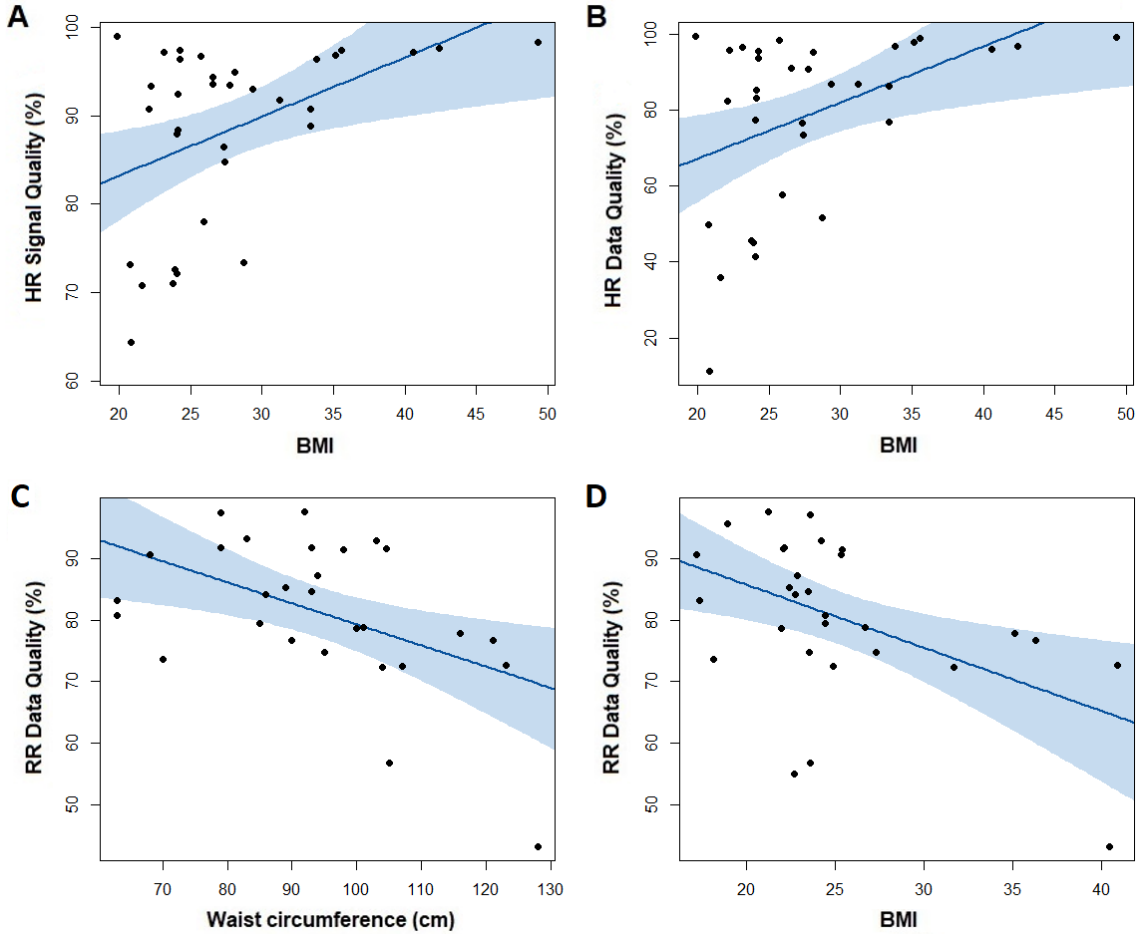
Associations within the Stable group (n=49) between (A) HR signal quality and BMI and (B) HR data quality and BMI, and associations within the AECOPD group (n=35) between (C) RR data quality and waist circumference and (D) RR data quality and BMI. The shaded area indicates 95% CI and *P* values and correlations calculated using Spearman ρ. AECOPD: acute exacerbation of chronic obstructive pulmonary disease; HR: heart rate; RR: respiratory rate.

For the Stable group, HR signal quality and HR data quality were positively correlated with BMI (*r*_s_=0.45, *P*=.008; *r*_s_=0.44, *P*=.008; Figures 3A and 3B). For the AECOPD group, RR data quality was negatively correlated with waist circumference and BMI (*r*_s_=–0.49, *P*=.009; *r*_s_=–0.44, *P*=.02; Figures 3C and 3D).

## Discussion

### Principal Findings

In this study, it was possible to continuously measure vital signs (RR, HR, ST, and PA) in free-living conditions using multiparameter wearable technology for people living with COPD after hospitalization and during stable symptoms. Measurement of vital signs using our technology was more challenging post AECOPD. The Equivital LifeMonitor was acceptable to some people with stable COPD symptoms and post AECOPD and produced data of sufficient quality. It should be noted that some participants felt uncomfortable with a device around their chest, and data quality was influenced by body composition. Overall, continuous VSM during daily life is possible, and its potential utility for supporting patient post AECOPD should be explored.

In this study, some participants stated that they did not want to wear a device around their chest. As well as the physical implications of a vest-like device, people with COPD may be influenced by the psychological impact of a device around the chest, as reported previously [26]. While most participants in our study reported no discomfort wearing the vest, similar to a previous study measuring respiratory rate with a chest belt [26], some participants reported that the vest felt restrictive at times or made them feel breathless. Perceived breathlessness could have influenced vest acceptability, as COPD populations tend to prefer watch-like devices [33]. However, measuring respiratory rate from such devices is challenging [34]. The use of a wearable, wireless patch has been successful in continuously monitoring vital signs of inpatients [35], and although this would reduce the perceived breathlessness with a vest-like device, Rubio et al [34] reported that a chest band could measure the RR more reliably than patch-like devices.

Recruitment was more challenging in the AECOPD group, with 15.4% of eligible patients recruited for the AECOPD group, compared to 41.3% in the Stable group, as seen in previous similar studies [9,17]. In a study where patients were asked to record oxygen saturation (SpO_2_) and RR using 2 separate devices for 2 months, 79% measured SpO_2_ and 60% measured RR three times per day, while 98% and 83% measured SpO_2_ and RR, respectively, once per day [26]. In this study, we observed a comparable adherence to findings when patients took daily measures (84% of participants provided vest data) but were able to capture a vast amount of data (on average 12 hours of data). Compared to the AECOPD group, a greater proportion of the Stable group wore the vest for >5 weeks (46% vs 71%). Technology continues to develop multiparametric wearable devices [36-39] to reduce patient burden, but it is possible that this remains a significant barrier in an AECOPD population with lower digital literacy.

Participant feedback from telephone calls suggested that the vest was acceptable overall, with 68% of participants reporting no problems. Some participants reported that they chose not to wear the vest on the days that they felt unwell. Vitacca et al [18] asked participants to complete a weekly 12-item Respicard (recording symptoms, SpO_2_, and HR) for 6 months and identified that participants with worse respiratory values had poorer adherence. In this study, 10% of the AECOPD group withdrew because they experienced problems wearing the vest, and 14% of participants in the AECOPD group struggled to engage with the device once returning home. Following discharge from hospital post AECOPD, symptoms remain elevated, and it takes time for patients to recover to their normal symptoms and daily activities [7,40]. Despite our single piece of technology reducing the need for patients to measure multiple vital signs and the observational nature of the study, the greater symptom burden in an AECOPD population reduced adherence to wearable technology.

Our results show that some patients were unable to participate as their chest size exceeded the maximum vest size, or they felt that the maximum vest size was not a suitable fitting. The Equivital LifeMonitor used in this study was originally designed to monitor vital signs in a military population [41]. A greater proportion of those who withdrew required a larger vest size than the completers (71% vs 34%). Existing wearable technology is more broadly marketed toward a healthy population and is typically not tailored for people living with COPD. While the form of the technology used in this study was generally acceptable, advancements in more discrete technologies are needed.

Similar to previous reports in healthy men [31], this study shows that HR and RR measurements obtained from the vest are of sufficient quality in a COPD population. Evidence suggests accurate vital signs measurements of clothing monitors such as the Zephyr BioHarness and Hexoskin [42,43], but our PPI members found such devices challenging to put on and remove owing to their tight-fitting nature. Despite the technology used in this study being tested by our PPI group, physical impairments affected participants’ ability to wear the vest and charge the SEM. Compared to 2% of the Stable group, 11% of the AECOPD group reported problems putting the SEM in the cradle, with some needing help from a cohabitant. The HR signal and data quality were also worse for people with a lower BMI, which is seen more often in an AECOPD population [44-46]. This may be owing to lower body composition and lower conductance [47]; therefore, a weaker connection between the skin and the electrodes embedded in the vest. These problems have been observed elsewhere [26] and must be considered by manufacturers, researchers, and clinicians when selecting digital health technologies.

### Limitations

Although the number of patients assessed in this study was low as a proportion of patients screened, introducing the possibility of selection bias, recruitment is often challenging post AECOPD [17,28,29]. Our single piece of technology aimed to passively capture multiple vital signs; however, some participants may prefer active participation to obtain recordings. It was not possible to measure SpO_2_ and blood pressure in the continuous and unobtrusive manner in line with this study. The lack of an age-matched healthy control group prevented us from identifying the unique difficulties with the use of multiparameter technology in a COPD population. This study may have benefitted from measuring vital signs overnight, to obtain individualized “baseline” vital sign values. A more rigorous qualitative exploration of participants’ experiences would have provided greater insights than telephone call field notes.

### Conclusions

Following hospitalization for AECOPD and during stable symptoms, it was possible to continuously measure RR, HR, ST, and PA using multiparameter wearable technology during free-living conditions. The Equivital LifeMonitor was acceptable to participants and produced data of sufficient quality, despite some reports of discomfort with wearing a device around the chest and data quality influenced by body composition. Overall, continuous VSM during daily life is possible for people living with COPD and its potential utility for supporting patients post AECOPD should be further explored.

## Appendix

Multimedia Appendix 1 Supplementary figures.

## Abbreviations

AECOPD: acute exacerbation of chronic obstructive pulmonary disease
COPD: chronic obstructive pulmonary disease
HR: heart rate
NHS: National Health Service
NIHR: National Institute for Health Research
PA: physical activity
PPI: patient and public involvement
PR: pulmonary rehabilitation
RR: respiratory rate
SEM: sensor electronics module
SpO_2_: oxygen saturation
ST: skin temperature
VSM: vital signs monitoring
