# Supplementary material for: Usability of Wearable Multiparameter Technology to Continuously Monitor Free-Living Vital Signs in People Living With Chronic Obstructive Pulmonary Disease: Prospective Observational Study
Source: JMIR Hum Factors. 2022 Feb 16;9(1):e30091. doi: 10.2196/30091 (PMC8892301; doi:10.2196/30091)
Supplement: Multimedia Appendix 1 [file humanfactors_v9i1e30091_app1.docx]

**Supplementary Figures**

**Figure S1:** Schematic timeline for the study protocol for AECOPD and Stable groups.

**
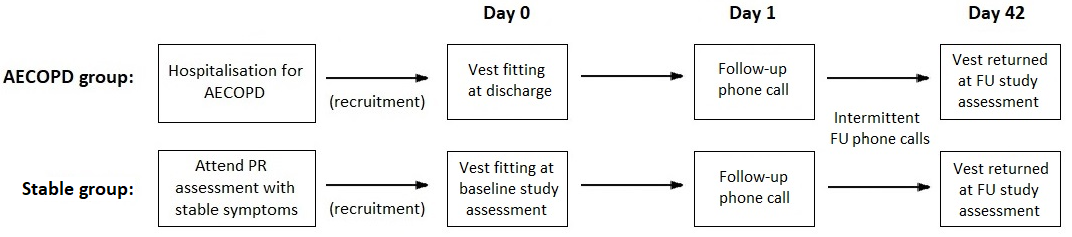
Abbreviations:** AECOPD: Acute Exacerbation of Chronic Obstructive Pulmonary Disease; FU: Follow-up; PR: Pulmonary Rehabilitation.

**Figure S2:** Comparisons between participants with the correct vest size and a larger vest size for; A) days worn; B) HR signal quality; C) HR data quality; D) wear time; E) RR signal quality; F) RR data quality. Data are shown as box plots composed of; the 25% percentile (the lower extremity of the box); the median (the central line of the box); and the 75% percentile (the upper extremity of the box). The lines outside each box correspond to the minimum and maximum values.

**
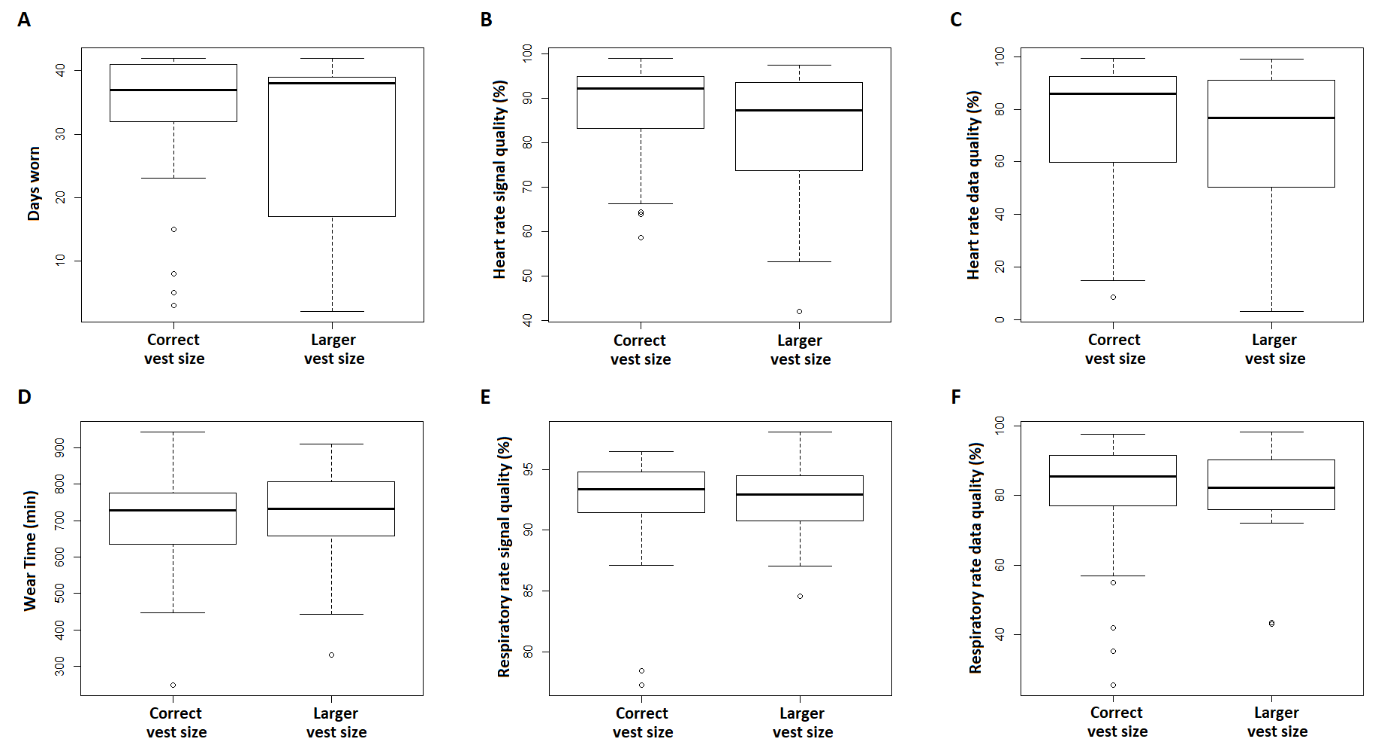
Abbreviations:** HR: Heart rate; RR: Respiratory rate.
